# The landscape of open science in behavioral addiction research: Current practices and future directions

**DOI:** 10.1556/2006.2023.00052

**Published:** 2023-10-05

**Authors:** Charlotte Eben, Beáta Bőthe, Damien Brevers, Luke Clark, Joshua B. Grubbs, Robert Heirene, Anja Kräplin, Karol Lewczuk, Lucas Palmer, José C. Perales, Jan Peters, Ruth J. van Holst, Joël Billieux

**Affiliations:** 1 Department of Experimental Psychology, Ghent University, Ghent Belgium; 2 Département de Psychologie, Université de Montréal, Montréal, Canada; 3 Louvain for Experimental Psychopathology Research Group (LEP), Psychological Sciences Research Institute, UCLouvain, Louvain-la-Neuve, Belgium; 4 Centre for Gambling Research at UBC, Department of Psychology, University of British Columbia, Vancouver, B.C., Canada; 5 Djavad Mowafaghian Centre for Brain Health, University of British Columbia, Vancouver, B.C., Canada; 6 Department of Psychology, University of New Mexico, Albuquerque, NM, USA; 7 Center for Alcohol, Substance Use, And Addiction (CASAA), University of New Mexico, Albuquerque, NM, USA; 8 School of Psychology, University of Plymouth, Plymouth, UK; 9 Faculty of Psychology, Technische Universität Dresden, Dresden, Germany; 10 Institute of Psychology, Cardinal Stefan Wyszynski University in Warsaw, Warsaw, Poland; 11 Department of Experimental Psychology; Mind, Brain and Behavior Research Center (CIMCYC), University of Granada, Granada, Spain; 12 Department of Psychology, Biological Psychology, University of Cologne, Cologne, Germany; 13 Department of Psychiatry, Amsterdam UMC -University of Amsterdam, Amsterdam, The Netherlands; 14 Center for Urban Mental Health, University of Amsterdam, Amsterdam, The Netherlands; 15 Institute of Psychology, University of Lausanne, Lausanne, Switzerland; 16 Center for Excessive Gambling, Addiction Medicine, Lausanne University Hospital (CHUV), Lausanne, Switzerland

**Keywords:** behavioral addiction, open science, reproducibility, gambling, gaming

## Abstract

Open science refers to a set of practices that aim to make scientific research more transparent, accessible, and reproducible, including pre-registration of study protocols, sharing of data and materials, the use of transparent research methods, and open access publishing. In this commentary, we describe and evaluate the current state of open science practices in behavioral addiction research. We highlight the specific value of open science practices for the field; discuss recent field-specific meta-scientific reviews that show the adoption of such practices remains in its infancy; address the challenges to engaging with open science; and make recommendations for how researchers, journals, and scientific institutions can work to overcome these challenges and promote high-quality, transparently reported behavioral addiction research. By collaboratively promoting open science practices, the field can create a more sustainable and productive research environment that benefits both the scientific community and society as a whole.

Although behavioral addiction research emerged at the end of the last century (Holden, 2001; Marks, 1990), the nosological status of a wide range of behavioral addictions (with the exception of Gambling and Gaming Disorders) remains debated (Billieux, Schimmenti, Khazaal, Maurage, & Heeren, 2015; Mihordin, 2012; Starcevic, Billieux, & Schimmenti, 2018). Globally, the field is still often considered as an “emerging” or “new” one. We decided to write this commentary to describe and evaluate the open science practices in the field of behavioral addictions to promote awareness and to encourage the field to adopt these practices to further improve research quality in this field. Our objective was to specify what we mean when talking about open science and identify the issues pertaining to the (perceived) *status quo* in the field of behavioral addictions regarding open science. It is worth acknowledging that we are not the first to call for more open and transparent research in this field, and therefore the current paper is oriented towards avenues and solutions to further integrate and promote open science.

## What is ‘open science’?

Open science (also referred to as open research and open scholarship) has been defined as: "An umbrella term reflecting the idea that scientific knowledge of all kinds, where appropriate, should be openly accessible, transparent, rigorous, reproducible, replicable, accumulative, and inclusive, all which are considered fundamental features of the scientific endeavor. Open science consists of principles and behaviors that promote transparent, credible, reproducible, and accessible science. Open science has six major aspects: open data, open methodology, open source, open access, open peer review, and open educational resources” (Elsherif, Flack, Kalandadze, Pennington, & Xiao, 2021). Thus, open science encompasses different practices across the life of a research project, as well as pre-registration of its design, hypotheses, and data analytic plan on an online platform or as Registered Report to have a time-stamped documentation before starting the project (Nosek et al., 2015; for further information see https://forrt.org/glossary/preregistration/).

These practices are essential to prevent well-established problems such as irreplicable and irreproducible research (i.e., failure to find the same result in a different sample using the same or similar methods [replicability] and being unable to find the same results using the same data [reproducibility]), the file drawer issue (i.e., statistically non-significant but important research that is not published and therefore remains hidden from the scientific community), and so-called ‘questionable research practices’ (i.e., problematic research practices that do not constitute ‘misconduct’, but are inconsistent with the principles of scientific integrity; for representative examples, see the next section; for comprehensive overviews see Korbmacher et al., 2023; Pennington, 2023a, 2023b).

Fostering the reliability and transparency of research results should reflect the core values of any research field, including that of behavioral addictions. Indeed, although behavioral addictions research has had a growing impact on international public health policies in recent years (e.g., recognition of Gaming Disorder by WHO, which contributed to the regulation of loot boxes and other gaming-related design features amplifying uncontrolled, and potentially addictive use; see Drummond, Sauer, Hall, Zendle, & Loudon, 2020; Flayelle et al., 2023), it has been criticized for largely not endorsing the current best practices in open science research (e.g., Grubbs, Floyd, Griffin, Jennings, & Kraus, 2022; van Rooij et al., 2018). Video gaming, gambling, online sexual activities, shopping, social networking and on-demand TV streaming are among the most popular non-substance-related leisure activities worldwide (Flayelle et al., 2023), and thus research on these behaviors has the potential to have a widespread impact on modern society. It seems imperative that experts and policy makers make impactful decisions based on transparently reported, reliable, and reproducible research.

In the following sections, we elaborate on the problems that arise from not practicing open science, the current status and challenges that the field of behavioral addictions is facing, and the opportunities and possible solutions that we foresee. Ultimately, open science practices such as sharing data or preregistering confirmatory, exploratory, and qualitative research can improve the quality of evidence that is used for important purposes, from policy making and education to prevention and treatment. Further, endorsing open science can also help to detect questionable research practices in this field.

## The problem

‘Questionable research practices’ include (but are not limited to) inappropriate sampling, questionable inferences, underpowered studies, p-hacking, hypothesizing after results are known (HARKing), unclear reporting of methods (e.g., missing information about time of data-collection, ambiguous data analysis plans), or salami slicing of data (i.e., not being sufficiently *transparent* about multiple use of the same data, or splitting a specific dataset in multiple papers; for example see John, Loewenstein, & Prelec, 2012). These practices are quite common across scientific disciplines (depending on the definition and assessment method, prevalence rates range from 15% to 51.2%; Gopalakrishna et al., 2022; Xie, Wang, & Kong, 2021), largely being attributable to current incentives in scientific publication that focus on quantitative indices of research impact (e.g., impact-factors of peer-reviewed journals, number of citations; for a discussion of a different incentive system see Schönbrodt et al., 2022) which do not correlate with the quality of research (Anderson, Ronning, De Vries, & Martinson, 2007; Dougherty & Horne, 2022; Higginson & Munafò, 2016). This incentive system does not sufficiently acknowledge the relevance of open science practices, in comparison to statistically significant and/or “novel” results. Consequently, many researchers lack the knowledge and/or motivation to apply open science practices (Nosek, Spies, & Motyl, 2012, 2015). This dynamic can have many negative consequences for the behavioral addictions field. For instance, a lack of data sharing can hinder cumulative science where researchers can combine and compare datasets (that are often hard to collect) across studies (see for example Pennington, 2023a, 2023b). This applies to direct and conceptual replication studies, but also to review projects and meta-analyses. Furthermore, shared data allows us to re-use and re-analyze already acquired data sets to generate or test new hypotheses. A lack of transparency could also mean that several studies reporting findings obtained from the same group of participants (i.e., salami slicing) count as cumulative samples in meta-analyses (see Hilgard, Sala, Boot, & Simons, 2019, for a critical account of this issue in relation to the positive effect of videogames on cognitive abilities). As a consequence of these questionable research practices, the reproducibility and validity of findings are jeopardized. The overall quality of the literature and knowledge about behavioral addictions are affected and we, as researchers, may see our scientific integrity as compromised. Open science practices, by contrast, can open up new research perspectives, such as data-driven commentary enabled through data sharing (for an example in the context of a Registered Report, see Amendola, 2023; Billieux & Fournier, 2023).

## The current status

In the field of clinical psychology, Tackett and Miller (2019), catalyzed earlier efforts to promote open science practices through their special section in the *Journal of Abnormal Psychology*. As the field of behavioral addiction is a subfield of clinical psychology, we will first summarize the work by Jennifer Tackett prior to focussing on the work done specifically in the field of behavioral addictions. In their initial work, Tackett et al. (2017) started the conversation about the replication crisis specifically in the field of clinical psychology which was extended in their later work (Tackett, Brandes, King, Markon, 2019). They discuss many open science practices and concerns within the clinical field regarding their use, as well as barriers and possible steps in their field to implementing open science practices. In another paper, Tackett, Brandes, and Reardon (2019) give specific advice on how to use Open Science Framework in the workflow of clinical psychological research and the related advantages.

Building on this work, in the behavioral addictions field, recent efforts have been made to identify why open science practices are not more commonly used and ways to tackle this issue. This is particularly the case in problem gambling research—the longest established domain of research related to behavioral addiction (Yau & Potenza, 2015). Research on problem gambling is thus not an “emerging” field, even if Gambling Disorder has only been recognized as an addictive disorder since 2013 when the DSM-5 (American Psychiatric Association, 2013) was released (it was previously conceptualized as an impulse-control disorder and diagnosed as such in the DSM-IV-TR and previous versions; American Psychiatric Association, 2000). Researchers in this field are increasingly aware that the adoption of open science practices is needed to improve research quality. In 2019, Wohl and colleagues announced that the field of gambling research was lagging behind other scientific fields in acknowledging the replication crisis. The authors called for more openness in the field, including study preregistration and reporting of power analyses, as well as replication studies.

In the wake of that initial article, other researchers proposed new directions for bridging the gap toward the implementation of open science practices in the field of gambling. For instance, Louderback, Wohl, and LaPlante (2021) suggested integrating open science practices with current guidelines for industry funded research in the gambling field. These authors argued that this dynamic could help to foster transparency and thus ensure independent industry funded research. Even though their paper specifically focuses on gambling research, these guidelines could easily be transposed to any industry-funded and non-industry-funded research (see Shi, Potenza, & Turner, 2020 for a similar approach in the field of gaming disorder).

Important new insights come from studies that have investigated knowledge of open science and its presence in the field of gambling research and video gaming. LaPlante, Louderback, and Abarbanel (2021) found that, not surprisingly, only a minority of gambling researchers (attending a 2019 conference) used open science practices, with many of them still having concerns and doubts on how to implement them. Among the most common concerns were privacy issues when sharing code, material, and data, and the fear that others would use the data code or materials without appropriate acknowledgement (LaPlante et al., 2021). Another study by Louderback et al. (2022) investigated the use of open science practices in a random sample of 500 gambling research articles. They found that open access publishing was the most used practice (35.2% of articles), while the use of other practices was very low (0–15%). Interestingly, these authors also observed that studies which adopted at least one open science practice received more citations than papers that did not adopt any open science practices (for similar observations in other fields, see Colavizza, Hrynaszkiewicz, Staden, Whitaker, & McGillivray, 2020; McKiernan et al., 2016; Piwowar & Vision, 2013; Wang, Liu, Mao, & Fang, 2015). Despite limited adoption of open science practices in the field to date, there is clear evidence of a change in perspective. As well as the above-mentioned articles by Wohl, Tabri, and Zelenski (2019) and Louderback et al. (2021), behavioral addiction researchers have called for preregistrations and Registered Reports of qualitative research in video gaming (Karhulahti, 2022), and more replication studies in the gambling field (e.g., Heirene, 2021; LaPlante, 2019).

Whilst the many calls for improved, open research are positive, the limited uptake of open science practices to date in the behavioral addiction field is concerning. As mentioned, research findings have the potential to directly influence the way policies are established, how education and prevention are conducted, and ultimately how patients are treated. Accordingly, as is the case for clinical psychology (Grubbs, 2022; Tackett, Brandes, & Reardon, 2019; Tackett, Brandes, King, et al., 2019) and mental health research more broadly, the risks of having irreplicable, inaccurate, or unclear research appear high for behavioral addiction research. There is an urgent need for behavioral addiction researchers to engage in open science practices to allow full and proper evaluations of their conclusions and to facilitate replications.

## The current challenges

The above-mentioned studies highlight that, although open-access publishing is widely adopted, our field is still lacking a satisfactory level of open and transparent research practices. One primary reason for this appears to be that researchers are not sufficiently educated about open science practices. Additional perceived barriers include the perception that using open science practices may take more time than the ‘traditional’ approach to conducting research, that shared datasets and materials may be used without acknowledgement, and that openly sharing parts of the research process (e.g., analysis code) may lead to criticism (Gownaris et al., 2022). In the following section we address some of these issues and propose possible ways to tackle them. After all, many of the barriers impeding open science practices can be addressed by appropriate transfer of knowledge about open science.

First of all, we would like to elaborate on the finding that open access publishing seems to be the most used open science practice (Gownaris et al., 2022; Louderback et al., 2022). On the one hand, we agree that open access publishing is crucial to largely and fairly distribute research results. Knowledge can only be used if there is access to it. Access may be more difficult for researchers at unaffiliated faculties or non-western universities, where access to expensive journals is limited. For example, some authors have argued that different types of resources (e.g., financial) and infrastructural deficiencies in non-western or non-economically developed societies hinder the generation and dissemination of knowledge in and from these societies (Au, 2007; Ross-Hellauer, 2022; Westwood & Jack, 2007). Open access publishing partly solves this problem and can easily be achieved: if a researcher or institution does not have the resources to choose the open access option of the journals provided, there is always the option to use green open access and publish a preprint on any preprint server for a citable time-stamped publication format. In order to make traditionally published papers available, the researcher can publish a post-acceptance version of the paper on an institutional or personal website. Information about the open access options and embargo periods for these options can be easily found on the Sherpa Romeo website (https://v2.sherpa.ac.uk/romeo/). On the other hand, in various countries, researchers pay to make their papers open access purely because they are required to. Many funders and countries require individual researchers to pay open access fees, otherwise they would be ineligible for future funding. But not every research institution has the ability to pay open access publishing fees, which can create a further gap between wealthy and less wealthy researchers and countries. Lastly, many so-called ‘Open Access’ publishers do not necessarily value good and open research practices. Many ‘predatory’ journals publish papers without a sufficiently rigorous peer-review process to profit from the open access fees. The concerning quality of such papers may in some cases contribute to creating negative attitudes towards open access publishing in general (Shen & Björk, 2015). Thus, we would question whether the adoption of open access publishing alone qualifies as adhering to open science principles. Perhaps more importantly, open science should refer to the process of science and not just the product of science. That is, openly sharing results via open access publications is a commendable final step in the scientific process, but open science can and should start long before the publication of results.

Second, we would like to address researchers' concerns that practicing open science takes more time, ultimately leading to less research output, which in turn could especially jeopardize the careers of early career researchers (for further information on open science in early career researchers [ECRs]) see Allen & Mehler, 2019). While we agree that initially starting to engage with open science practices is demanding, this does not necessarily equate to less research output. More to the point, given that all results produced during the process of research can and should be considered outputs (i.e., published protocols, citable datasets, published papers, replicable analytical code), practicing open science might lead to more output because each aspect of the research process demonstrates a researcher's productivity and also provides an output that future researchers may use and acknowledge via citation. Moreover, preregistration and especially Registered Reports can facilitate the publication of null results. This can be a boon to burgeoning researchers for whom, in times past, may have had to relegate such results to their file draw, resulting in nothing more than wasted time and effort.

## The solutions

The above concerns and objections, though very real, are hardly new and are not exclusive to behavioral addictions research. Clinical psychological science has been grappling with issues around the implementation of open science principles for several years (Tackett, Brandes, King, et al., 2019; Tackett et al., 2017). As such, there are already important recommendations for clinical psychologists and mental health researchers that address many of the above concerns (e.g., Tackett, Brandes, & Reardon, 2019). Flowing from such recommendations, below we have outlined a number of suggestions and insights for behavioral addictions research more broadly.

Most importantly, implementing open science principles is not an ‘all-or-nothing’ process; rather, it can better be described as a ‘buffet approach’ wherein researchers might choose what works best for them (for a discussion of the ‘buffet approach’ see Bergmann, 2023). When starting to implement open science in your workflow, it can be overwhelming to keep track of which practices are considered to be useful and how to implement them. As such, if you are a researcher who wants to address the above-mentioned issues, we would advise starting with practices for which you can see a clear benefit for you and the field, and which are easiest to implement in your current workflow. For example, perhaps your data is already fully anonymized and you have the consent of participants to share the data due to a standard consent form. In such a case, sharing your data alongside your publication might be an ideal first step. Another possibility is to start with writing a detailed preregistration. In such cases, the time spent writing up the hypotheses and methods before collecting data is time saved when completing an ethics application and when writing the manuscript after collecting data. Thus, study preregistration does not necessarily take more time, it just shifts *when* in the project you spend this time on writing (for a similar discussion see Heirene et al., 2021). Importantly, preregistration does not prevent or prohibit you from performing further (transparent) exploratory analysis (Höfler, Scherbaum, Kanske, McDonald, & Miller, 2022). As in these examples, it is possible to implement open science in small steps into your workflow and if everybody only changes one habit, we can make a difference in the field. Therefore, leading-by-example can arguably be our most powerful tool to make a change in the field as single researchers.

Moving beyond individual changes we can make as researchers, there are also individual changes we can make as scientists within our fields. In the peer review process, referees can directly ask and encourage authors to share data and materials, and add the “standard reviewer disclosure request”, if necessary (https://osf.io/hadz3/). Furthermore, while we applaud journal policies that mandate data sharing, there is a concern that some journals do not follow up on these policies such that data sharing is not always enforced (Gabelica, Bojčić, & Puljak, 2022). In this case, the community can urge journals to implement *and enforce* the adoption of open science practices by providing open science badges after verification or making data, code and material sharing mandatory (Thibault, Pennington, & Munafò, 2023). There is a growing number of networks and repositories, such as the Open Science Framework and national reproducibility networks which support sharing practices. However, to prevent “openwashing” (e.g. provide supposedly open data or code that is not understandable to others), it is important to at least randomly verify these materials. This process could also benefit from making requirements for data sharing transparent. For example, journals could specify information that needs to be included as accompanying information, such as Readme files that details specific information on each column of the data file. Furthermore, journals publishing behavioral addiction research should more often enable Registered Reports as an article type. To our knowledge, specifically in the field of behavioral addictions, only the journals *Addiction Research and Theory* and *Psychology of Addictive Behaviors* offer “Registered Reports” as an article type to date (see Karhulahti et al., 2022; Grubbs et al., 2022 for examples in these two journals). Registered Reports help to evaluate the importance of a research question and the suitability of the design to answer this particular question before data collection begins, which can save time and effort in getting the work published after data collection (Chambers, Feredoes, Muthukumaraswamy, & Jetchells, 2014; Chambers & Tzavella, 2022; Nosek & Lakens, 2014).

Another form of publishing that might address some of our concerns mentioned above is publishing (Registered Reports) via the Peer Community In (PCI; https://peercommunityin.org/pci-and-journals/), which describes a standardized review process for preprints. After peer-review via PCI, preprints become valid and citable articles. These articles can usually stay on a preprint server without being published in journals, but have still gone through a thorough peer-review process. Alternatively, these articles can be published in the Peer Community Journal as it is, immediately, and at no costs. One last option for publication is to submit articles which received an in-principle-acceptance by PCI Registered Report (PCI RR) to PCI friendly journals. These journals typically immediately accept the PCI RR accepted article without further review. Lastly, there are also PCI RR-interested journals which may consider the in-principle-accepted version but provide no commitment (for further information see https://rr.peercommunityin.org/about/pci_rr_friendly_journals and https://rr.peercommunityin.org/about/pci_rr_interested_journals). Because of this, open access publishing is possible without any costly open access fees and PCI recommended articles are increasingly recognized by many scientific commissions (for more information on this publication type see Chambers & Tzavella, 2022; Pennington & Heim, 2022).

The entire behavioural addictions community can take action as well. For example, to transfer knowledge as a community, we can use conferences, teaching, and workshops to inform about the replication crisis and the value of transparency and replication, focusing on long-term benefits for the students and the field. For example, Louderback et al. (2022) suggest that ECRs especially can make a positive change for the field, but only if knowledge about open science practices is taught. Here, we encourage the readers to have a look at the website of the Framework for Open and Reproducible Research Training (FORRT; https://forrt.org/). The site provides many useful resources to support teaching open and reproducible research practices (Parsons et al., 2022; Pownall et al., 2021).

When starting a PhD program, it might be particularly helpful and impactful to reflect on the usefulness of adopting certain open science practices. For example, a replication attempt of the most impactful study for the project could be a good starting point (for a discussion see Wagge et al., 2019) as well as preregistration in our projects, again in accordance with the ‘buffet-approach’: adopt what works best for you. This can also reduce the perceived stress experienced by PhD students who might otherwise hunt for significant results. Here we urge the senior researchers among our readers to support the efforts of their ECRs to practice open science. Graduate programs could mandate open science practices as the default for a PhD dissertation (e.g., at least one data chapter should include a preregistered protocol; or that a justification must be provided if data cannot be uploaded to a university archive). Moreover, it would be useful to pay more attention to the quality of research when evaluating CVs and achievements rather than focusing on the number of publications and significant results. This approach is in line with the idea of ‘slow science’ which focuses on the quality, transparency, and rigor of research projects rather than the simple output in the form of a publication (Frith, 2020). Again, in our opinion other research outputs such as code, data and materials should also be considered valid outputs in the evaluation process. This approach, however, requires a shift in our current (publication-oriented) incentive system by funders, publishers, governments, and institutions (Stewart et al., 2022; for such an incentive system see e.g., the DORA declaration: https://sfdora.org/).

Lastly, as a community we can and should take larger-scale efforts to tackle issues of lacking high-powered replications and generally poorly powered studies. Data sharing and combined efforts can lead to ‘multi-lab’ approaches as they have done in cognitive and social psychology (i.e., the ‘many labs’ efforts, the Psychological Science Accelerator efforts, or the Reproducibility Project: Psychology initiated by the Center for Open Science), but also in the field of compulsive sexual behaviors/problematic pornography use recently (i.e., International Sex Survey; Bőthe et al., 2021; but see Pennington, Jones, Tzavella, Chambers, & Button, 2022, for the promotion of such efforts in addiction research in general). A key component of these approaches is that several labs and institutions involved combine their efforts to collect bigger and more diverse samples using high-quality research methods. A similar approach can be used to conduct replication studies that can achieve the statistical power required to reliably support the presence or absence of an effect (Heirene, 2021). Decision-making around *which* studies or effects we should try to replicate can be taken as a research community. Here we suggest approaches such as an expert consensus of key effects, or a systematic approach as described in Isager et al. (2021; for further discussion on which studies deserve replication attempts see Heirene, 2021). With large-scale, community efforts we can not only start tackling the issue of poorly powered (replication) studies, but also combine our efforts in an efficient way to identify the studies that deserve a replication attempt most.

To support the transition to a more open and transparent behavioral addictions field, we have summarized our recommendations in Table 1.

**Table 1.** Opportunities to increase open science practices in the field and their actors

What?	Who?
Start with what works best for you	Individual
Preregister the study as preregistration or (PCI) Registered Report	Individual
Ensure that participants agree and sign the informed consent that allows data sharing	Individual
Share data and materials on a public repository	Individual
Publish open access or post the work on a public preprint server or institutional website	Individual
Ask authors to share data, code, and/or materials in the peer review process	Individual
Urge journals to implement open science practices	Individual/community
Graduate programs mandating/encouraging open science practices	Institution
Offer funding schemes for academics to use or enhance their open science toolkit	Institution/funding bodies
Adopt the idea of ‘slow science’	Institution/funding bodies/community
Embedding replication and open science practices in PhD programs	Institution
Enforce the journal open science policies	Journals
Offer registered report options	Journals
Transfer knowledge	Community
Evaluate quality of research not quantity (in grant reviews and reviews of individuals)	Community/funding bodies/institution
Evaluate researchers on a broad range of research contributions and outputs	Community
Take larger scale replication efforts	Community

## Conclusion

In conclusion, we believe that if the behavioral addictions research community sends the signal that we value more open, transparent and reproducible research, we will be able to achieve this. It will require a step-by-step community effort — each person must change their day-to-day research habits to align with the principles of open science; we must educate the next generation of researchers sufficiently so that conducting open, transparent research is the norm. In doing so, we can transition behavioral addictions research towards a more transparent, reliable, and reproducible field. Ultimately, this will allow us to obtain a more fine-grained understanding of processes underlying behavioral addictions and to develop effective prevention programs and clinical interventions for those experiencing them.

## Funding sources

This work was supported by an ERC Consolidator grant awarded to Frederick Verbruggen (PI of CE; European Union's Horizon 2020 research and innovation programme, grant agreement No 769595).

## Authors' contribution

CE initiated the idea of writing this opinion piece. JB helped to make contact with all authors. CE and JB wrote the initial draft. BB, DB, LC, JBG, RH, AK, KL, LP, JCP, JP, and RJV gave input on the topics and provided critical revisions on the manuscript. All authors approved the final version.

## Conflict of interest

JB is associate editor for the Journal of Behavioral Addictions. LC has served as an associate editor of JBA in 2022. LC is the Director of the Centre for Gambling Research at UBC, which is supported by funding from the Province of British Columbia and the British Columbia Lottery Corporation (BCLC), a Canadian Crown Corporation. The Province of BC government and the BCLC had no role in the preparation of this manuscript, and impose no constraints on publishing. LC has received a speaker/travel honorarium from the International Center for Responsible Gaming (US) and Scientific Affairs (Germany), and has received fees for academic services from the International Center for Responsible Gaming (US), GambleAware (UK), Gambling Research Exchange Ontario (Canada) and Gambling Research Australia. He has not received any further direct or indirect payments from the gambling industry or groups substantially funded by gambling. RH worked for the Gambling Treatment and Research Centre at the University of Sydney from 2019 to 2021 where his work was partially funded by Responsible Wagering Australia, an organization representing multiple Australian online wagering operators. He has not received any further direct or indirect payments from the gambling industry. RJV currently serves as associate editor of JBA and has not received any direct or indirect payments from the gambling industry or groups substantially funded by gambling.
